# Supplementary material for: Combining lineage correlations and a small molecule inhibitor to detect circadian control of the cell cycle
Source: iScience. 2025 Mar 22;28(4):112269. doi: 10.1016/j.isci.2025.112269 (PMC12002663; doi:10.1016/j.isci.2025.112269)
Supplement: Document S1. Figures S1–S5, Tables S1–S14, and Methods S1–S10 [file mmc1.pdf]

iScience, Volume *28*

Supplemental information

Combining lineage correlations and a small molecule inhibitor to detect circadian control of the cell cycle

Anjoom Nikhat, Arsh Shaikh, and Shaon Chakrabarti

Contents

S1 Methods S1: Model 1 – A simplified gene network model of circadian clock - cell cycle coupling, related to Figure 1. **2**

S2 Methods S2: Model 2 – Description of the coupled circadian clock - cell cycle model, related to Figure 1. **5**

1. Simplified Circadian clock model 5
2. Coupling the circadian clock - cell cycle system 5

S3 Method S3: Model 3 – Description of coupled circadian clock and cell cycle model, related to Figure 1. **8**

1. Complex cell cycle network 8
2. WEE1 mediated circadian clock forward coupling to cell cycle 9
3. p21 mediated circadian clock forward coupling to cell cycle 10

S4 Method S4: Numerical Integration of the system of ODEs to generate time series: incorporating constant Gaussian noise, related to Figure 1 and 2. **12**

S5 Method S5: Numerical Integration of the system of ODEs to generate time series : using the Chemical Langevin Equations (CLE), related to Figure 1 and 2. **13**

S6 Method S6: Fourier Analysis of time series data, related to Figure 2. **13**

S7 Method S7: Simulation to generate cellular lineages, related to Figure 1 and 2. **14**

S8 Method S8: Simulating KL001 mediated circadian clock inhibition, related to Figure 3. **20**

S9 Method S9: Digital extraction of KL001 data and downstream processing, related to Figure 3. **22**

S10 Method S10: The Ergodic Principle and detecting M phase oscillations in cellular lineages, related to Figure 5. **22**

S1 Methods S1: Model 1 – A simplified gene network model of circadian clock - cell cycle coupling, related to Figure 1.

Since only phenomenological models have so far been used to explain the emergence of the cousin-mother inequality in cell cycle time correlations, here we explored whether a very simple network model of the circadian clock coupled to the cell cycle [1] is sufficient to generate lineage correlations. The circadian clock network used in [1], which we describe as Model 1, consists only of the PER-CRY regulatory loop. Model 1 includes the BMAL1-CLOCK mediated activation of transcription of the *Per* and *Cry* mRNAs. *Per* or *Cry* isoforms are not explicitly considered, but instead clubbed together as a single species. The mRNAs are translated in the cytoplasm and upon translocation back to the nucleus they inhibit their own production. This system is represented by a set of 7 coupled ODEs (Equations 1-7).

The mammalian cell cycle is driven by multiple Cyclin and Cyclin-dependent Kinases. In this simplified model the authors however consider the entire cell cycle to be dependent on the concentration of CyclinB-Cdk1 complex or the Mitosis Promoting Factor(MPF) [1]. Progression through each of these phases is dependent on certain concentration thresholds of MPF. The cells in G1 phase enter the S-phase when [MPF] $>$ 0.09 nM. The model also includes a tyrosine kinase WEE1 which inhibits Cdk1 and prevents the G2/M transition. Hence for cells to enter the M-phase the [MPF] must be greater than that of [WEE1]. Once [MPF] surpasses [WEE1] another inhibitor of MPF is activated which is analogous to the Anaphase Promoting Complex (APC). This inhibitor brings the [MPF] down to a lower threshold ($\leq$ 0.06 nM) for the cell to exit mitosis and enter G1 phase. This system is represented by a set of 3 coupled ODEs (Equations 8-10).

The circadian clock mediated forward coupling was included via BMAL1-CLOCk mediated control of kinase protein WEE1 of the cell cycle, thus resulting in control of G2/M transition by the clock. The strength of this coupling is controlled by the value of the coupling constant C1. The variable names corresponding to each species in Model 1 are mentioned in Table S1. The variables associated with circadian clock are described by y_i, $i = 1, 2, ..., 7$ and those associated with the cell cycle are indicated by z_j, $j = 8, 9, 10$. The parameter values for Model 1 are mentioned in Tables S2 and S3. We numerically integrated the Chemical Langevin form [2] of the set of 10 ordinary differential equations that constituted the model as described later in Section 3.

The control of the autonomous cell cycle period (TCC) in this model was imposed by multiplying the RHS of the 3 cell cycle ODEs by a scaling factor of (97.4/TCC). For Model 1, TCC = 16 hrs.

Variable	Molecular species
y_1	PER-CRY mRNA
y_2	PER-CRY cytoplasmic protein
y_3	PER-CRY nuclear protein
y_4	BMAL1 mRNA
y_5	BMAL1 cytoplasmic protein
y_6	BMAL1 nuclear protein
y_7	BMAL1-CLOCK complex
z_8	Mitosis Promoting Factor (MPF)
z_9	WEE1
z_{10}	MPF-inhibitor (APC)

Table S1: Molecular species and corresponding variable names for Model 1. (Related to Figure 1)

$$\frac{dy_1}{dt} = \frac{v_{1b}.(y_7 + c)}{k_{1b}.(1 + (\frac{y_3}{k_{1i}})^p) + (y_7 + c)} - k_{1d}.y_1 \tag{1}$$

$$\frac{dy_2}{dt} = k_{2b}.{y_1}^q - k_{2t}.y_2 + k_{3t}.y_3 - k_{2d}.y_2 \tag{2}$$

$$\frac{dy_3}{dt} = k_{2t}.y_2 - k_{3t}.y_3 - k_{3d}.y_3 \tag{3}$$

$$\frac{dy_4}{dt} = \frac{v_{4b}.y_3^r}{k_{4b}^r + y_3^r} - k_{4d}.y_4 \tag{4}$$

$$\frac{dy_5}{dt} = k_{5b}.y_4 - k_{5t}.y_5 + k_{6t}.y_6 - k_{5d}.y_5 \tag{5}$$

$$\frac{dy_6}{dt} = k_{5t}.y_5 - k_{6t}.y_6 + k_{7a}.y_7 - k_{6a}.y_6 - k_{6d}.y_6 \tag{6}$$

$$\frac{dy_7}{dt} = k_{6a}.y_6 - k_{7a}.y_7 - k_{7d}.y_7 \tag{7}$$

$$\frac{dz_8}{dt} = (\frac{k_{0mpf}.k_{1mpf}^{n1}}{k_{1mpf}^{n1} + z_8^{n1} + s.z_{10}^{n1}}).(z_8^{max} - z_8) - d_{wee1}.z_9.z_8 \tag{8}$$

$$\frac{dz_9}{dt} = (\frac{1}{k_{actw} + d_{w1}}).(c_w + C_1.(y_7 - y_7^{inh})) + (\frac{k_{actw}}{k_{actw} + d_{w1}} - 1).(\frac{k_{inactw}.z_8^{n1}.z_9}{k_{1wee1}^{n1} + z_8^{n1}}) - d_{w2}.z_9 \tag{9}$$

$$\frac{dz_{10}}{dt} = k_{act}.(z_8 - z_{10}) \tag{10}$$

In Eq. (9), the c_w term prevents the production rate of z_9 (Wee1) from going negative. The minimum concentration of BMAL1-CLOCK (y_7) is ~ 0.8 and even for the maximum coupling strength considered, that is $C1 = 1.5$, the term $C1(y_7 - y_7^{inh})$ is equal to -0.24. Since the value of c_w or the constant activator is equal to 1.46 (Table S3), the production term will always remain positive.

Parameter Symbol	Numerical Value
c	$0.01nM$
p	8
v_{1b}	$9nMh^{-1}$
k_{1b}	$1nM$
k_{1d}	$0.12h^{-1}$
k_{1i}	$0.56nM$
k_{2b}	$0.3nM^{-1}h^{-1}$
k_{2d}	$0.05h^{-1}$
k_{2t}	$0.24h^{-1}$
k_{3t}	$0.02h^{-1}$
q	2
k_{3d}	$0.12h^{-1}$
v_{4b}	$3.6nMh^{-1}$
r	3
k_{4b}	$2.16nM$

Parameter Symbol	Numerical Value
k_{4d}	$0.75h^{-1}$
k_{5b}	$0.24h^{-1}$
k_{5d}	$0.06h^{-1}$
k_{5t}	$0.45h^{-1}$
k_{6t}	$0.06h^{-1}$
k_{6d}	$0.12h^{-1}$
k_{6a}	$0.09h^{-1}$
k_{7a}	$0.003h^{-1}$
k_{7d}	$0.09h^{-1}$

Table S2: Circadian clock ODE parameters for Model 1. (Related to Figure 1)

Parameter Symbol	Numerical Value
k_{0mpf}	$10h^{-1}$
k_{1mpf}	$0.05nM$
$n1$	2
s	50
d_{wee1}	$5nM^{-1}h^{-1}$
k_{actw}	$1h$
d_{w1}	$1h$
k_{inactw}	$200h^{-1}$
k_{1wee1}	$0.5nM$
d_{w2}	$1h^{-1}$
k_{act}	$0.01h^{-1}$
c_w	$1.46nM$
y_7^{inh}	$0.9629nM$
z_8^{max}	$1nM$

Table S3: Cell Cycle ODE parameter values for both Model 1 and Model 2. (Related to Figure 1)

S2 Methods S2: Model 2 – Description of the coupled circadian clock - cell cycle model, related to Figure 1.

1. Simplified Circadian clock model

Our circadian clock network in Model 1 only included the PER-CRY mediated negative regulatory loop as mentioned in Section 1, allowing introduction of a forward coupling from the circadian clock to the cell cycle. To allow the reverse interaction, i.e. from the cell cycle to the circadian clock, we introduced a second loop in the circadian clock network where the BMAL1-CLOCK complex also activates the production of *Rev-erb*α mRNA (Model 2). The REV-ERBα protein on translocation back to the nucleus gives rise to oscillations in the concentration of the BMAL1 protein. Decreasing concentration of both PER-CRY complex and the REV-ERBα protein lifts the inhibition causing active BMAL1-CLOCK complex to accumulate, thus resetting the cycle. The modified circadian clock in Model 2 is described by 10 ODEs as shown below (Equations 11 to 20).

The cell cycle network was the same for Model 1 and Model 2 (Equations 8-10), hence the thresholds used to transition cells through the various cell cycle stages remain the same.

2. Coupling the circadian clock - cell cycle system

The circadian clock mediated forward coupling is as described before, via the control of cell cycle protein WEE1 by which the circadian clock prevents the G2/M transition [3]. To incorporate the reverse control of the cell cycle on the circadian clock, we incorporated Cdk1 mediated inhibition of REV-ERBα [4]. Since we do not explicitly have Cdk1 in our cell cycle system, MPF was used as the inhibitory link. The strengths of the forward and the reverse couplings are controlled by the coupling constants C_1 and C_2 respectively in the equations for WEE1 and REV-ERBα proteins.

The reactions were modelled using a combination of Michaelis-Menten kinetics, along with first and second order reaction kinetics. The 10 ODEs for the circadian clock model along with the 3 ODEs describing the cell cycle as in Section 1, together depict the time evolution of the concentrations of different molecular species included as shown in Figure 1A in the main text. The variable names of the species in Model 2 are mentioned in Table S4 and the parameter values for the system of 10 ODEs is provided in Table S5.

Variable	Molecular species
y_1	PER-CRY mRNA
y_2	PER-CRY cytoplasmic protein
y_3	PER-CRY nuclear protein
y_4	BMAL1 mRNA
y_5	BMAL1 cytoplasmic protein
y_6	BMAL1 nuclear protein
y_7	BMAL1-CLOCK complex
y_8	REV-ERBα mRNA
y_9	REV-ERBα cytoplasmic protein
y_{10}	REV-ERBα nuclear protein

Table S4: Molecular species and corresponding variable names for the Circadian clock network in Model 2. (Related to Figure 1)

$$\frac{dy_1}{dt} = v_{sp}.(\frac{y_7^n}{k_{ap}^n + y_7^n}) - k_{1d}.y_1 \tag{11}$$

$$\frac{dy_2}{dt} = k_{2b}.{y_1}^q - k_{2t}.y_2 + k_{3t}.y_3 - k_{2d}.y_2 \tag{12}$$

$$\frac{dy_3}{dt} = k_{2t}.y_2 - k_{3t}.y_3 - k_{3d}.y_3 \tag{13}$$

$$\frac{dy_4}{dt} = v_{sb}.(\frac{k_{ib}^m}{k_{ib}^m + y_{10}^m}) - k_{4d}.y_4 \tag{14}$$

$$\frac{dy_5}{dt} = k_{5b}.y_4 - k_{5t}.y_5 + k_{6t}.y_6 - k_{5d}.y_5 \tag{15}$$

$$\frac{dy_6}{dt} = k_{5t}.y_5 - k_{6t}.y_6 + k_{7a}.y_7 - k_{6a}.y_6 - k_{6d}.y_6 \tag{16}$$

$$\frac{dy_7}{dt} = k_{6a}.y_6 - k_{7a}.y_7 - k_7.y_7.y_3 - k_{7d}.y_7 \tag{17}$$

$$\frac{dy_8}{dt} = v_{sr}.(\frac{y_7^h}{k_{ar}^h + y_7^h}) - k_{8d}.y_8 \tag{18}$$

$$\frac{dy_9}{dt} = k_{9b}.y_8 - k_{9t}.y_9 + k_{10t}.y_{10} - k_{9d}.y_9 - C_2.v_{cdk1}.z_8.(\frac{y_9}{k_p + y_9}) \tag{19}$$

$$\frac{dy_{10}}{dt} = k_{9t}.y_9 - k_{10t}.y_{10} - k_{10d}.y_{10} - C_2.v_{cdk1}.z_8.(\frac{y_{10}}{k_p + y_{10}}) \tag{20}$$

In order to achieve an autonomous circadian clock period (TCR) of 24 hrs, we multiply the 10 ODEs representing the circadian clock gene network by a factor of 1.45 and the autonomous cell cycle period (TCC) is imposed as described in Section 1. For the forward coupled system the TCC = 15 hrs and for the reverse coupled case TCC = 16 hrs.

Parameter Symbol	Numerical Value
v_{sp}	$1.5nMh^{-1}$
k_{ap}	$0.7nM$
k_{1d}	$0.7h^{-1}$
n	2
k_{2b}	$0.3nM^{-1}h^{-1}$
q	2
k_{2d}	$0.05h^{-1}$
k_{2t}	$0.24h^{-1}$
k_{3t}	$0.02h^{-1}$
k_{3d}	$0.12h^{-1}$
v_{sb}	$1.8nMh^{-1}$
m	2
k_{ib}	$2.2nM$
k_{4d}	$0.4h^{-1}$
k_{5b}	$0.24h^{-1}$
k_{5d}	$0.06h^{-1}$
k_{5t}	$0.45h^{-1}$
k_{6t}	$0.06h^{-1}$
k_{6d}	$0.12h^{-1}$
k_{7a}	$0.003h^{-1}$
k_{6a}	$0.09h^{-1}$
k_{7d}	$0.09h^{-1}$
k_7	$1nM^{-1}h^{-1}$
v_{sr}	$1.6nMh^{-1}$
k_{ar}	$0.6nM$
h	2
k_{8d}	$0.2h^{-1}$
k_{9b}	$1.7h^{-1}$
k_{9t}	$0.8h^{-1}$
k_{10t}	$0.4h^{-1}$
k_{9d}	$0.2h^{-1}$
k_{10d}	$0.2h^{-1}$
v_{cdk1}	$1h^{-1}$
k_p	$1.006nM$

Table S5: Circadian clock ODE parameter values for Model 2. (Related to Figure 1)

S3 Method S3: Model 3 – Description of coupled circadian clock and cell cycle model, related to Figure 1.

1. Complex cell cycle network

The circadian clock model used is the same as in Model 2. It includes the PER-CRY based negative feedback loop and REV-ERBα protein mediated control of BMAL1 protein. The mammalian cell cycle is driven by a network of Cyclin and Cyclin-dependent kinases. The previous cell cycle model was driven purely by CyclinB/Cdk1 complex or MPF. In order to simulate a more realistic scenario we implemented a more complex cell cycle model whose variables included all the Cyclin/Cdk complexes previously suggested in literature [5]. The variables and the equations associated with the cell cycle network are as follows:

Variable	Molecular species
z_1	cyclinD/Cdk4-6 complex
z_2	transcription factor E2F
z_3	cyclinE/Cdk2 complex
z_4	cyclinA/Cdk2 complex
z_5	cyclinB/Cdk1 complex
z_6	Anaphase-promoting complex

Table S6: Molecular species and corresponding variable names for the complex cell cycle model. (Related to Figure 1)

$$\frac{dz_1}{dt} = v_{sd}\cdot\frac{GF}{k_{gf}+GF} - v_{dd}\cdot\frac{z_1}{k_{dd}+z_1} \tag{21}$$

$$\frac{dz_2}{dt} = v_{1e2f}\cdot\frac{(E2F_{tot}-z_2)}{k_{1e2f}+(E2F_{tot}-z_2)}.(z_1+z_3) - v_{2e2f}\cdot\frac{z_2}{k_{2e2f}+z_2}.z_4 \tag{22}$$

$$\frac{dz_3}{dt} = v_{se}.z_2 - v_{de}.z_4\cdot\frac{z_3}{k_{de}+z_3} \tag{23}$$

$$\frac{dz_4}{dt} = v_{sa}.z_2 - v_{da}.z_6\cdot\frac{z_4}{k_{da}+z_4} \tag{24}$$

$$\frac{dz_5}{dt} = v_{sb1}.z_4 - v_{db}.z_6\cdot\frac{z_5}{k_{db}+z_5} \tag{25}$$

$$\frac{dz_6}{dt} = v_{1cdc20}.z_5\cdot\frac{(Cdc20_{tot}-z_6)}{k_{1cdc20}+(Cdc20_{tot}-z_6)} - v_{2cdc20}\cdot\frac{z_6}{k_{2cdc20}+z_6} \tag{26}$$

The parameter values for this version of the model are given in the table below:

Parameter Symbol	Numerical Value
v_{sd}	$0.175 nMh^{-1}$
k_{gf}	$0.1 nM$
GF	$1 nM$
v_{dd}	$0.245 nMh^{-1}$
k_{dd}	$0.1 nM$
v_{1e2f}	$0.805 h^{-1}$
$E2F_{tot}$	$3 nM$
k_{1e2f}	$0.01 nM$
v_{2e2f}	$0.7 h^{-1}$
k_{2e2f}	$0.01 nM$
v_{se}	$0.21 h^{-1}$
v_{de}	$0.35 h^{-1}$
k_{de}	$0.1 nM$
v_{sa}	$0.175 h^{-1}$
v_{da}	$0.245 h^{-1}$
k_{da}	$0.1 nM$
v_{sb1}	$0.21 h^{-1}$
v_{db}	$0.28 h^{-1}$
k_{db}	$0.005 nM$
v_{1cdc20}	$0.21 h^{-1}$
$Cdc20_{tot}$	$5 nM$
k_{1cdc20}	$1 nM$
v_{2cdc20}	$0.35 nMh^{-1}$
k_{2cdc20}	$1 nM$

Table S7: Parameters for Complex cell cycle model. (Related to Figure 1)

2. WEE1 mediated circadian clock forward coupling to cell cycle

We first checked if the results obtained using the Wee1 mediated coupling for the simpler model holds true for the complex model as well and hence modified the set of equations in the following way to incorporate the same. The additional variables and the modified equations are as follows:

Variable	Molecular species
z_7	Wee1 mRNA
z_8	Wee1 protein

Table S8: New variables included to incorporate Wee1 mediated forward coupling. (Related to Figure 1)

$$\frac{dz_1}{dt} = v_{sd}.\frac{GF}{k_{gf} + GF} - v_{dd}.\frac{z_1}{k_{dd} + z_1} \tag{27}$$

$$\frac{dz_2}{dt} = v_{1e2f}.\frac{(E2F_{tot} - z_2)}{k_{1e2f} + (E2F_{tot} - z_2)}.(z_1 + z_3) - v_{2e2f}.\frac{z_2}{k_{2e2f} + z_2}.z_4 \tag{28}$$

$$\frac{dz_3}{dt} = v_{se}.z_2 - v_{de}.z_4.\frac{z_3}{k_{de} + z_3} \tag{29}$$

$$\frac{dz_4}{dt} = v_{sa}.z_2 - v_{da}.z_6.\frac{z_4}{k_{da} + z_4} \tag{30}$$

$$\frac{dz_5}{dt} = v_{sb1}.z_4 - v_{db}.z_6.\frac{z_5}{k_{db} + z_5} - v_{m2b}.z_8.\frac{z_5}{k_{2bi} + z_5} \tag{31}$$

$$\frac{dz_6}{dt} = v_{1cdc20}.z_5.\frac{(Cdc20_{tot} - z_6)}{k_{1cdc20} + (Cdc20_{tot} - z_6)} - v_{2cdc20}.\frac{z_6}{k_{2cdc20} + z_6} \tag{32}$$

$$\frac{dz_7}{dt} = c_w + v_{sw}.C1.\frac{y_7^{nmw}}{k_{aw}^{nmw} + y_7^{nmw}} - v_{dmw}.\frac{z_7}{k_{dmw} + z_7} \tag{33}$$

$$\frac{dz_8}{dt} = k_{sw}.z_7 - v_{m7b}.z_5.\frac{z_8}{k_{7b} + z_8} - k_{dwee1}.z_8 \tag{34}$$

The strength of the coupling is controlled by changing the value of C1. Hence if C1 = 0 then the circadian clock and the cell cycle networks are uncoupled. The parameter values listed in Table S7 were utilized during the integration process, along with additional parameters specific to this version of the model, as detailed in the table below.

Parameter Symbol	Numerical Value
v_{m2b}	$2.1h^{-1}$
k_{2bi}	$0.1nM$
c_w	$0.012nMh^{-1}$
v_{sw}	$2.5nMh^{-1}$
k_{aw}	$2nM$
nmw	4
v_{dmw}	$0.5nMh^{-1}$
k_{dmw}	$0.5nM$
k_{sw}	$5h^{-1}$
v_{m7b}	$1.2h^{-1}$
k_{7b}	$0.1nM$
k_{dwee1}	$0.1h^{-1}$

Table S9: Extra parameters to incorporate Wee1 mediated forward coupling. (Related to Figure 1)

3. p21 mediated circadian clock forward coupling to cell cycle

The complex cell cycle network we implemented enabled us to test alternative coupling mechanisms mediated by molecular species other than Wee1. We therefore next explored p21 based forward coupling. The p21 protein acts as a Cdk-inhibitor and therefore delays the cell cycle duration. The synthesis of p21 is regulated in a circadian manner by REV-ERBα. REV-ERBα impedes the production of p21 and hence unlike Wee1 mediated coupling where the circadian clock slows down the cell cycle, by controlling p21 concentration, the circadian clock speeds up the cell cycle by inhibiting a Cdk-inhibitor.

The variables and equations used to incorporate p21 coupling in the network are as follows:

Variable	Molecular species
z_9	p21 mRNA
z_{10}	p21 protein
z_{11}	p21-CyclinE/Cdk2 inhibitor complex
z_{12}	p21-CyclinA/Cdk2 inhibitor complex
z_{13}	p21-CyclinB/Cdk1 inhibitor complex

Table S10: New variables included to incorporate p21 mediated forward coupling. (Related to Figure 1)

$$\frac{dz_1}{dt} = v_{sd}.\frac{GF}{k_{gf} + GF} - v_{dd}.\frac{z_1}{k_{dd} + z_1} \quad (35)$$

$$\frac{dz_2}{dt} = v_{1e2f}.\frac{(E2F_{tot} - z_2)}{k_{1e2f} + (E2F_{tot} - z_2)}.(z_1 + z_3) - v_{2e2f}.\frac{z_2}{k_{2e2f} + z_2}.z_4 \quad (36)$$

$$\frac{dz_3}{dt} = v_{se}.z_2 - v_{de}.z_4.\frac{z_3}{k_{de} + z_3} - k_{c3}.z_3.z10 + k_{c4}.z_{11} \quad (37)$$

$$\frac{dz_4}{dt} = v_{sa}.z_2 - v_{da}.z_6.\frac{z_4}{k_{da} + z_4} - k_{c5}.z_4.z10 + k_{c6}.z_{12} \quad (38)$$

$$\frac{dz_5}{dt} = v_{sb1}.z_4 - v_{db}.z_6.\frac{z_5}{k_{db} + z_5} - k_{c7}.z_5.z10 + k_{c8}.z_{13} \quad (39)$$

$$\frac{dz_6}{dt} = v_{1cdc20}.z_5.\frac{(Cdc20_{tot} - z_6)}{k_{1cdc20} + (Cdc20_{tot} - z_6)} - v_{2cdc20}.\frac{z_6}{k_{2cdc20} + z_2}.z_6 \quad (40)$$

$$\frac{dz_9}{dt} = v_{s1p21b} - C2.v_{smp21}.\frac{k_{ip21}^{nmp21}}{k_{ip21}^{nmp21} + y_{10}^{nmp21}} - v_{dmp21}.\frac{z_9}{z_9 + k_{dmp21}} \quad (41)$$

$$\frac{dz_{10}}{dt} = v_{s1p21}.z_9 - k_{c3}.z_3.z10 + k_{c4}.z_{11} - k_{c5}.z_4.z10 + k_{c6}.z_{12} - k_{c7}.z_5.z10 + k_{c8}.z_{13} - k_{ddp21}.z_{10} \quad (42)$$

$$\frac{dz_{11}}{dt} = k_{c3}.z_3.z_{10} - k_{c4}.z_{11} \quad (43)$$

$$\frac{dz_{12}}{dt} = k_{c5}.z_4.z_{10} - k_{c6}.z_{12} \quad (44)$$

$$\frac{dz_{13}}{dt} = k_{c7}.z_5.z_{10} - k_{c8}.z_{13} \quad (45)$$

The strength of the coupling is controlled by changing the value of C2. Hence if C2 = 0 then the circadian clock and the cell cycle networks are uncoupled. The parameter values listed in Table S7 were utilized during the integration process, along with additional parameters specific to this version of the model, as detailed in the table below.

Parameter Symbol	Numerical Value
v_{s1p21b}	$0.01nMh^{-1}$
v_{smp21}	$0.46nMh^{-1}$
k_{ip21}	$0.05nM$
$nmp21$	1
v_{dmp21}	$10nMh^{-1}$
k_{dmp21}	$0.5nM$
v_{s1p21}	$50h^{-1}$
k_{c3}	$0.2nM^{-1}h^{-1}$
k_{c4}	$0.1h^{-1}$
k_{c5}	$0.15nM^{-1}h^{-1}$
k_{c6}	$0.125h^{-1}$
k_{c7}	$0.12nM^{-1}h^{-1}$
k_{c8}	$0.2h^{-1}$
k_{ddp21}	$0.08h^{-1}$

Table S11: Extra parameters to incorporate p21 mediated forward coupling. (Related to Figure 1)

S4 Method S4: Numerical Integration of the system of ODEs to generate time series: incorporating constant Gaussian noise, related to Figure 1 and 2.

The system of ODEs that represent the coupled circadian clock-cell cycle gene network for both Model 1 and Model 2 can be evolved to generate time series data representing the change in concentration of various proteins and mRNAs. We numerically integrate the ODEs, using the Euler method which states that a differential equation of the form,

$$\frac{dx}{dt} = f(x)$$

can be discretized as follows:

$$\frac{x(t+\Delta t) - x(t)}{\Delta t} = f(x) \tag{46}$$

The ODEs are deterministic in nature, however owing to the inherent stochasticity in the biological system we incorporated gaussian noise into this model to generate stochastic differential equations (SDEs) and integrated them using the Euler-Maruyama scheme:

$$x_i(t+\Delta t) = x_i(t) + f(\vec{x})\Delta t + A\sqrt{\Delta t}N(0,1) \tag{47}$$

where x_i represents the ith molecular species in the models above (either circadian clock or cell cycle components), $\vec{x}$ represents the vector of all molecular species, and the term $A.N(0,1).\sqrt{\Delta t}$ is the Gaussian noise term added. Here A is the noise coefficient that is set by comparing Inter-mitotic times distribution as described in section 5, and $N(0,1)$ is a Gaussian Random variable with mean 0 and standard deviation 1.

S5 Method S5: Numerical Integration of the system of ODEs to generate time series : using the Chemical Langevin Equations (CLE), related to Figure 1 and 2.

In the previous section we introduced Gaussian noise in order to generate the SDEs. We also implemented the CLE framework in order to include state-dependent noise and integrated them using the Euler-Maruyama scheme [2]:

$$x_i(t+\Delta t) = x_i(t) + f(\vec{x})\Delta t + A\sum_{j=1}^{m} v_{i,j}\sqrt{a_j}N(0,1) \tag{48}$$

where x_i denotes the concentration of the ith species (circadian clock or cell cycle), $v_{i,j}$ is the net stoichiometric change in the ith species as a result of the jth reaction, and a_j represents the propensity of the j_{th} reaction. $N(0,1)$ represent a Gaussian random variable with mean 0 and standard deviation 1. We scale the noise in our simulations using the parameter denoted by A.

While in the main text we have included results when simulations were run using constant Gaussian noise, we implemented the state-dependent noise framework and obtained the observed correlations for the different models mentioned above (Figures S3 and S4).

S6 Method S6: Fourier Analysis of time series data, related to Figure 2.

To study the phenomenon of entrainment, we utilised Fourier Transforms to generate the spectral density *versus* frequency curves. This allowed us to detect the distribution of frequencies that constitute the oscillatory time series data for the network (say cell cycle) that is being entrained by the other network (say circadian clock) and vice versa. We fed the time series after eliminating the initial transients into the `spectrum` function in the R programming language with parameter values as follows: span = 5, log = ”no”, plot = FALSE. The default frequency axis for this function is in cycles per sampling interval. It is more intuitive to express the frequency axis in cycles per unit time hence we divide the extracted frequencies by our sampling rate. We also multiplied the spectral density by 2 so that the area under the periodogram equals the variance of the time series[6]. Thus for time series data say X, we run the following block of code to generate the spectral density curves mentioned in the main text (Figure 2) and SI (Figures S2 and S3). These curves demonstrate the phenomenon of entrainment where increasing coupling strengths changes the period of an oscillator closer to the period of the oscillator entraining it.

```
fourier_x <- spectrum(X,log="no",span=5,plot=FALSE)
freq_x <- fourier_x$freq/sampling_rate
spectral_x <- 2*fourier_x$spec
plot(freq_x,spectral_x,xlab="frequency",ylab="spectral density",type="l")
```

S7 Method S7: Simulation to generate cellular lineages, related to Figure 1 and 2.

The system of SDEs (constant noise or state-dependent noise) for either models were used to develop a simulation that creates cellular lineages where the progression through cell cycle is mediated by the different protein concentrations. We utilized the IGRAPH package in R to represent the growing population as a set of directed graphs [7, 8]. After initialisation of a set of ancestral cells which formed the base nodes of the graph, we assigned starting concentrations of the molecular species to each ancestral cell. We then numerically integrated the SDEs separately for each cell in order to determine the concentrations in the next time steps.

For Model 1 and Model 2 that uses the simpler cell cycle model depending upon concentration of Mitotis Promoting factor(MPF), the cells traverse the different cell cycle phases and the cells that ultimately reach the M-phase divide to give two daughter cells. The thresholds of MPF concentration for the different transitions used in the simulation are as follows:

Transition	Threshold
G1 to S/G2	[MPF] $\geq$ 0.09nM
G2 to M	[MPF] $>$ [Wee1]
M to G1 (division)	[MPF] $\leq$ 0.06nM

Table S12: MPF concentration threshold used in lineage simulation. (Related to Figure 1)

For Model 3 where we have implemented a complex cell cycle model, the following thresholds govern the progression through the cell cycle:

Transition	Threshold
$G1 \rightarrow S$	[cyclinA/Cdk2] $>$ [CyclinE/Cdk2]
$S \rightarrow G2$	[cyclinA/Cdk2] peaks
$G2 \rightarrow M$	[cyclinB/Cdk1] $>$ [CyclinA/Cdk2]
$M \rightarrow G1(division)$	[cyclinD/Cdk4-6] $>$ [CyclinB/Cdk1]

Table S13: Thresholds governing transitions through various cell cycle phases. (Related to Figure 1)

Upon reaching the M-phase, we remove the mother cell from the population and add two new nodes (representing two daughter cells) to the lineage graph. The daughter cells enter the G1 phase with concentrations of all species identical to that of the mother cell and the simulation runs till a user-defined final number of cells in the population are reached. The lineage simulation is schematised in Figure S1.

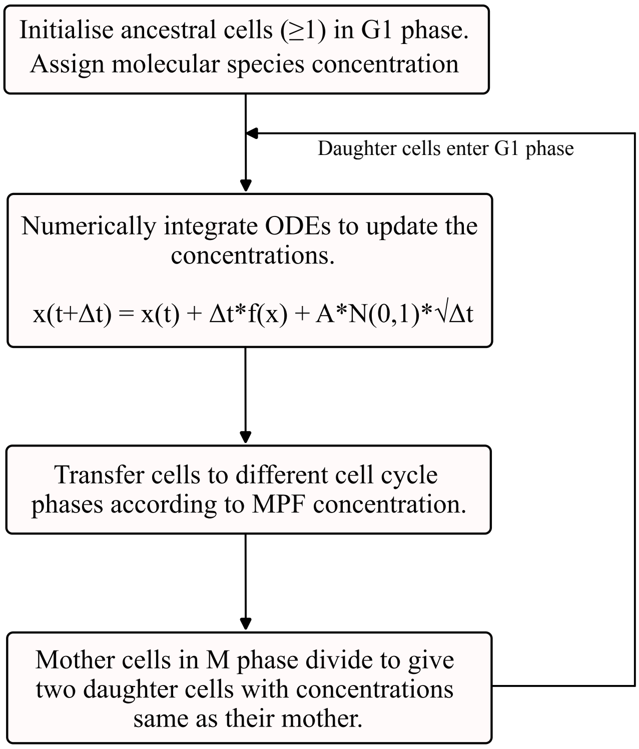

Figure S1: Outline of lineage algorithm. (Related to Figure 1)

A single run of this simulation generates lineage trees for the starting ancestor cells. From these directed lineage graphs we extracted the inter-mitotic times of each cell as (`division_time - birth_time`). The noise parameter A in the SDEs of either model (Equation 60 and 61) is optimized by matching the simulated IMT distribution with experimental data on HCT116 cells [9]. This parameter was set to the following values for the different cases:

Model	Noise version included	Noise scaling parameter (A) value
Model 1	Constant gaussian noise	0.0025
Model 2	Constant gaussian noise	0.003
Model 2	State-dependent noise	0.015
Model 3 (Wee1 coupled version)	State-dependent noise	0.04
Model 3 (p21 coupled version)	State-dependent noise	0.06

Table S14: Noise scaling parameters values for the different simulation runs. (Related to Figure 1)

We extracted unique related cell pairs like sisters, cousins, mother-daughter pairs and determined the Pearson correlation for the IMTs of each pair. Thus one lineage simulation generated a set of correlation values for each type of pair considered indicated by ρ_{SS} for sister correlation, ρ_{CC} for cousin correlation and ρ_{MD} for Mother-Daughter correlation respectively. For each run we also obtained the proliferation rate of the population by fitting a linear model to the $\log(cell\_number)$ versus time plot using the `fit.lm` function in R, after excluding the initial transients. The slope of the fit is the population proliferation rate.

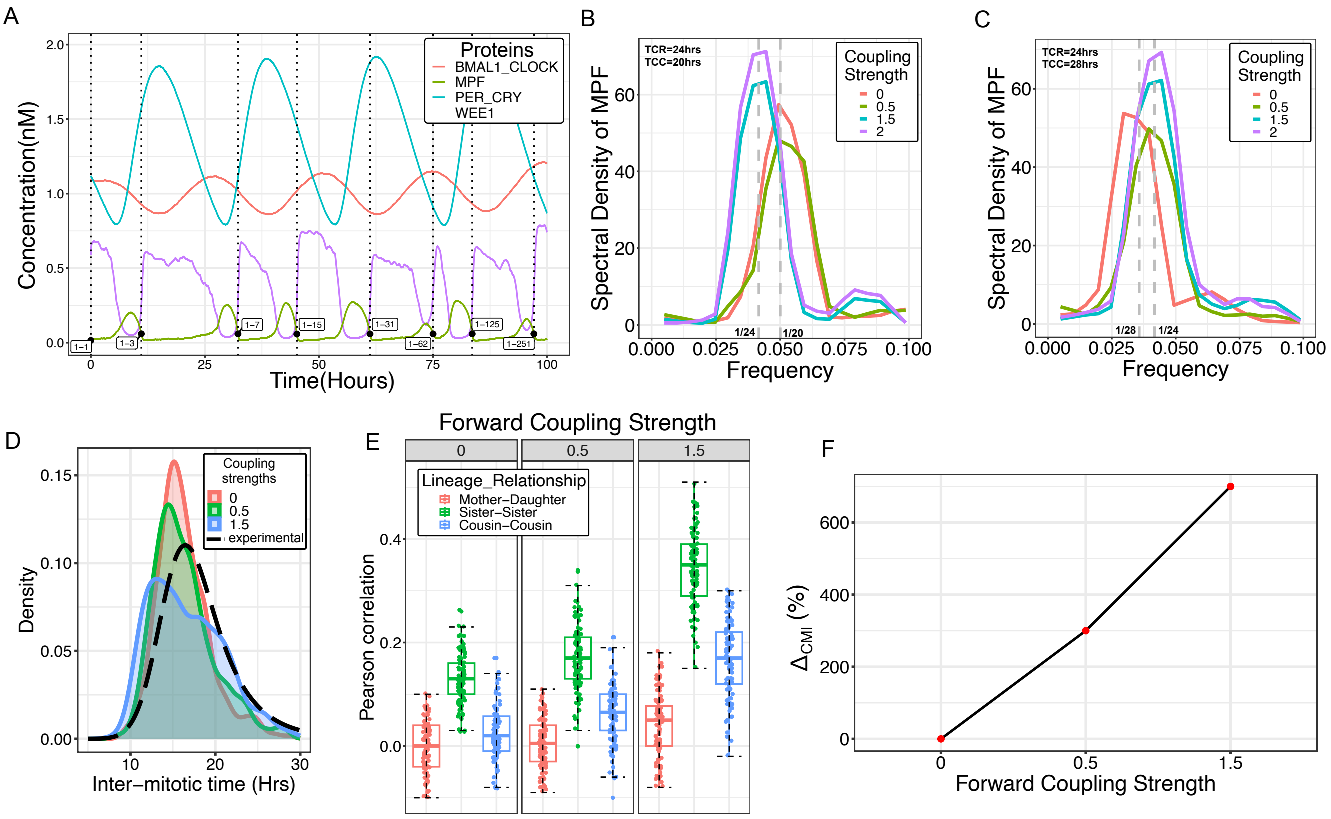

Figure S2: Forward coupled circadian clock – cell cycle gene network correlates Inter-mitotic times in cellular lineages for Model 1: (A) Time evolution of different proteins for a lineage, recapitulating the expected oscillations. The cells belonging to a lineage that were tracked to generate these oscillatory trajectories are labelled at the time of their birth on the graph. The different proteins present in the network satisfy the experimentally observed phase relationships. (B) & (C) Entrainment of cell cycle by the circadian clock. Increasing forward coupling strength changes the cell cycle frequency from 1/20 or 1/28 hour^{-1} respectively to the circadian clock frequency of 1/24 hour^{-1}. (D) With increasing forward coupling strength the distribution of the Inter-Mitotic times (demonstrated by the different colours) match the experimentally observed distribution. (E) The emergence of high sister correlation and also cousin-mother inequality with increasing coupling strength as seen in Model 2. (F) Percentage change in the difference between the median value of cousins and mother-daughter correlation (i.e. cousin-mother inequality) in comparison to the uncoupled system, shown for the different coupling strengths considered for the forward case. (Boxplot and median value calculated for 100 runs of simulation. Here TCC=16hrs). (Related to Figure 1 and 2)

For a fixed parameter set (denoted by i), We ran a set of 100 simulations and generated a distribution of Pearson correlation values for sisters (ρ_{SS}), cousins (ρ_{CC}) and mother-daughter pairs (ρ_{MD}). From this distribution we calculated the cousin-mother inequality for this parameter set as follows:

$$\rho^i_{CMI} = \text{median}(\rho_{CC}) - \text{median}(\rho_{MD})$$

We calculated ρ^i_{CMI} for different parameter sets (for example by changing the coupling strength or KL001 concentration) and then calculated the percentage change in cousin-mother inequality for each parameter set i compared to a control parameter set, Δ^i_{CMI}:

$$\Delta^{i}_{CMI}\% = \frac{(\rho^{(i)}_{CMI} - \rho^{(control)}_{CMI})}{\rho^{(control)}_{CMI}} * 100$$

Therefore when i represents the control set of parameters, $\Delta^{i}_{CMI}\%$ is 0. In case of simulations with different coupling strengths, similar to the behaviour of Model 2 (main text Figure 2), we observed emergence of the cousin-mother inequality for increasing coupling strengths in Model 1 and Model 3 as well (Figure S2 and S4).

For each of the 100 simulations that we ran for a particular parameter set, we also calculated the proliferation rate of the population to generate a distribution of proliferation rates. The percentage change in proliferation rate $\Delta_{growth}\%$ with respect to a control set of parameters is then similarly calculated as for the cousin-mother inequality described above. If the median population growth rate for the 100 runs of the simulation for the ith parameter set is indicated as $GR^{(i)}$ then $\Delta_{growth}\%$ is calculated as:

$$\Delta_{growth}\% = \frac{(GR^{(i)} - GR^{(control)})}{GR^{(control)}} * 100.$$

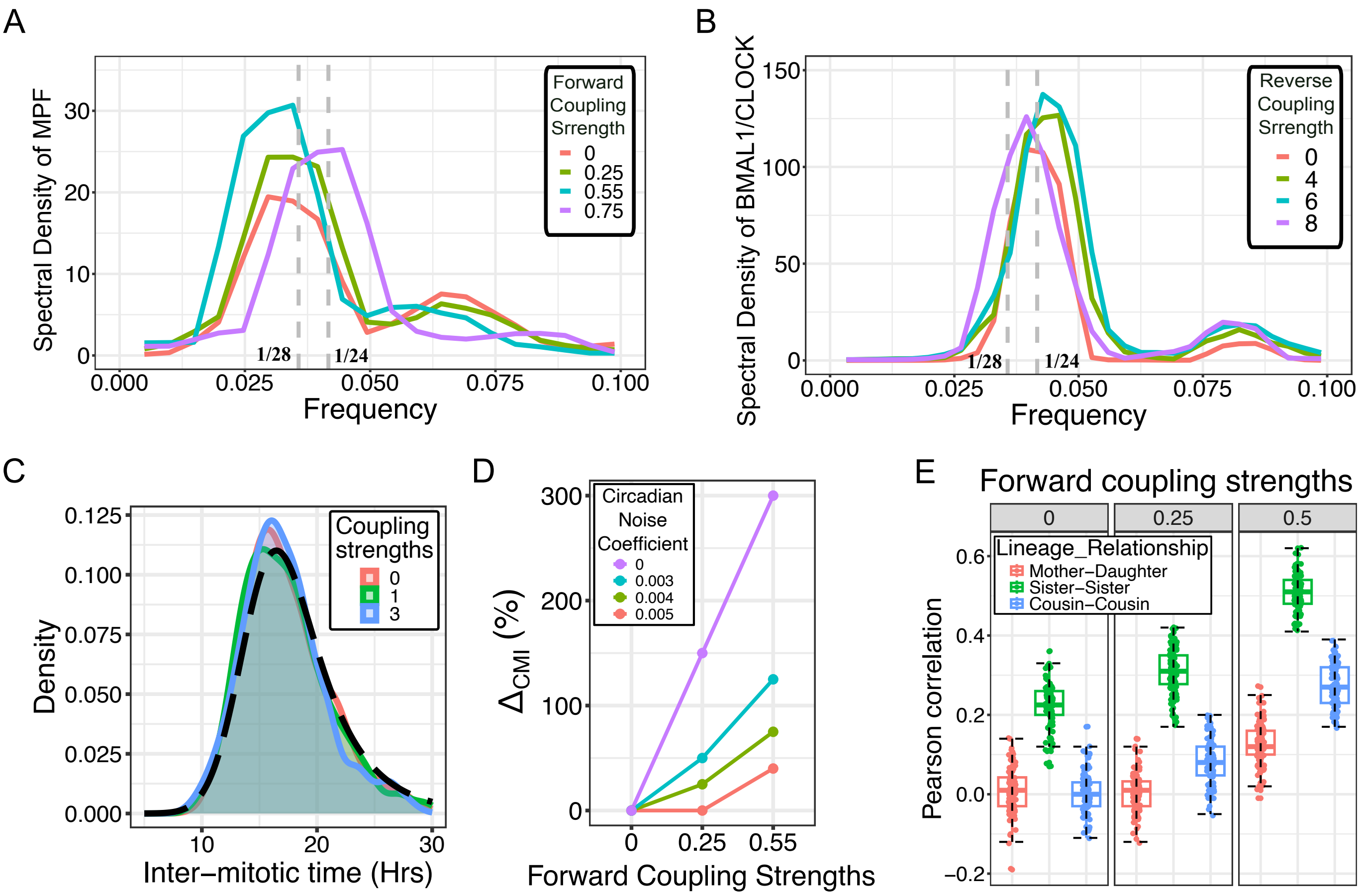

Figure S3: Simulation results for Model 2. (A)-(D) implement constant Gaussian noise while (E) implements state-dependent noise. (A) Entrainment of the cell cycle by circadian clock. With increasing forward coupling strength the frequency of oscillation of cell cycle gene MPF changes from 1/28 to 1/24 hour^{-1}. (B) Entrainment of circadian clock by the cell cycle. With increasing reverse coupling strength the circa- dian clock heterodimer BMAL1/CLOCK oscillation frequency changes from 1/24 to 1/28 hour^{-1}. Circadian clock period (TCR)=24 hrs, Autonomous cell cycle period (TCC)=28 hrs. (B) Comparison of IMT distribution from simulation and experiment for a reverse coupled system. The simulated IMT distribution matches the experimentally observed IMT distribution (black dashed line). (D) Effect of incorporating different noise coefficients for the circadian clock while keeping the noise coefficient for cell cycle fixed to what is mentioned in Section 5. With increasing noise in the circadian clock gene network the percentage change in cousin mother inequality $\Delta_{CMI}\%$ for C1 $\geq$ 0 when compared to uncoupled system that is C1 = C2 = 0, decreases. (E) Lineage simulation implemented with state-dependent noise where the noise scaling parameter is equal to 0.015. We observed that consistent to addition of constant Gaussian noise, the cousin-mother inequality emerged only when the system included forward coupling that is the circadian clock mediated control of the cell cycle. (Related to Figure 2)

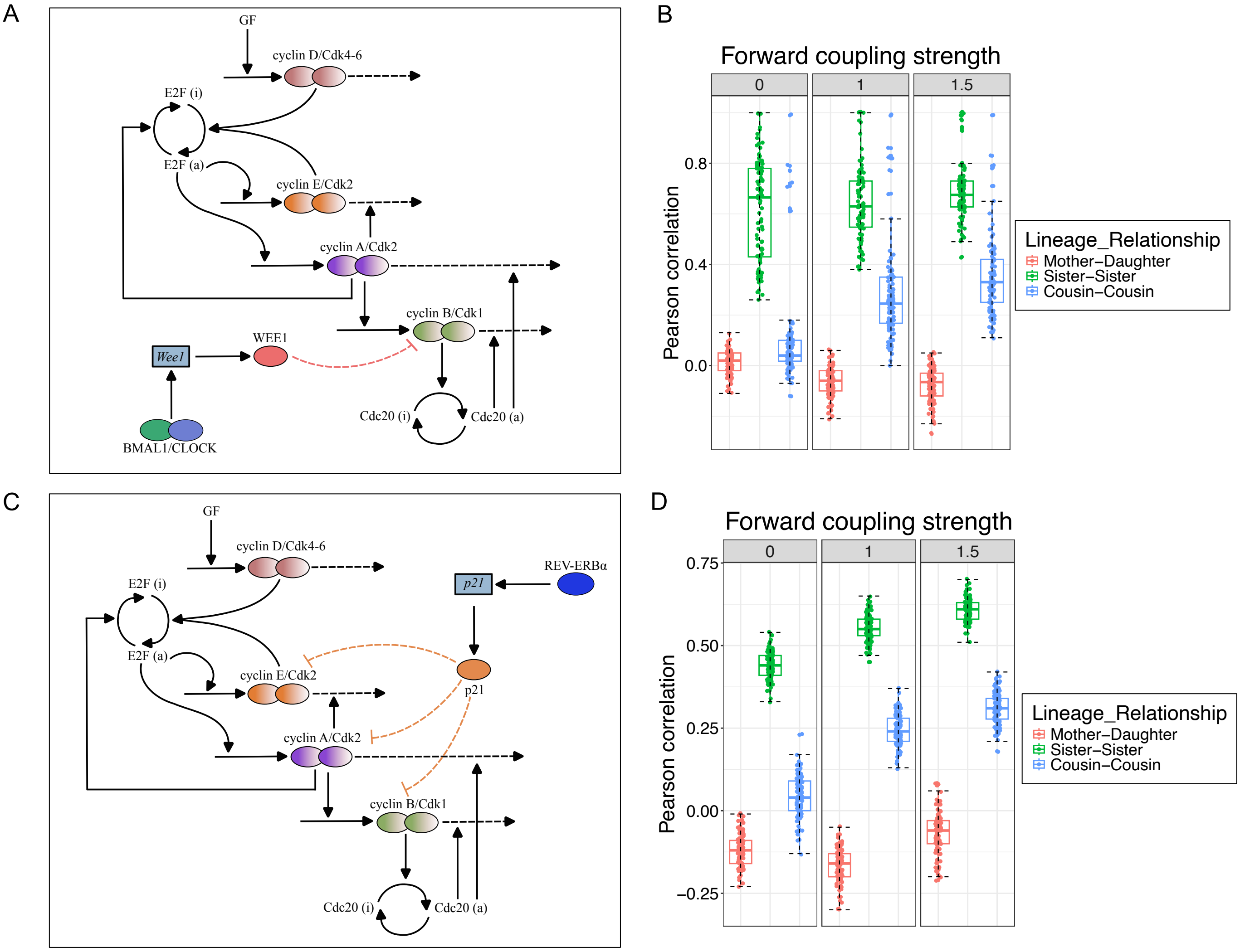

Figure S4: Forward coupled circadian clock – cell cycle gene network correlates Inter-mitotic times in cellular lineages for Model 3. All panels were generated with state-dependent noise. (A) Schematic of the more complex cell cycle network (Model 3). The model contains four main cyclin/Cdk complexes, the transcription factor E2F and the protein Cdc20. We also included the WEE1 protein which allows us to incorporate the BMAL1-CLOCK complex mediated circadian activation of the WEE1 protein. At the beginning of the cell cycle. At the beginning of the cell cycle, the growth factor GF ensures the synthesis of the CyclinD/Cdk4-6 complex which promotes progression through G1 phase. This complex activates the trnascription factor E2F, which then brings about the activation of CyclinE/Cdk2 and CyclinA/Cdk2 which are responsible for G1/S transition, S phase progression and the S/G2 transition. CyclinA/Cdk2 activates the synthesis of CyclinB/Cdk1 which leads to G2/M transition. During mitosis, CyclinB/Cdk1 activates protein Cdc20 which degrades the various Cyclin/Cdk complexes thus leading to exit from mitosis and resetting of the cycle. To include circadian control we incorporated BMAL1-CLOCK mediated activation of WEE1 kinase which inhibits CyclinB/Cdk1 thus delaying G2/M transition. (B) The emergence of cousin-mother inequality with increasing forward coupling strength when the lineage simulation was governed by the gene network described in A. (C) The complex cell cycle network which includes the Cdk inhibitor p21. p21 protein inhibits CyclinE/Cdk2, CyclinA/Cdk2 and CyclinB/Cdk1 thereby prolonging the cell cycle duration. The circadian control is imposed via the clock protein REV-ERBα which inhibits p21 and accelerates the cell cycle. (D) The emergence of cousin-mother inequality with increasing forward coupling strength when the lineage simulation was governed by the gene network described in C. (Related to Figure 2)

S8 Method S8: Simulating KL001 mediated circadian clock inhibition, related to Figure 3.

In order to mimic the effect of KL001 mediated circadian clock inhibitor, we decreased the degradation rates of the PER-CRY complex [10]. KL001 prevents ubiquitin - mediated degradation of the CRY proteins. Previous studies have shown that the degradation occurs both in cytoplasm as well as nucleus, hence we divided both the degradation rates k_{2d} and k_{3d} by the numbers mentioned in the graphs in main text Figure 3 as well as SI Figure S4 to incorporate KL001's effect. Dividing by 1 is the control, where the degradation rates are unchanged and hence inhibition is absent. Division by higher numbers reduces the degradation rates, and represents higher KL001 concentrations. The percentage change in cousin-mother inequality and proliferation rate for the different KL001 inhibition cases were determined as mentioned in section 5. For Model 1 we observed a decrease in the $\Delta_{CMI}\%$ for higher KL001 values (> 1) similar to the behaviour observed for Model 2 in main text Figure 3. However unlike in Model 2, where the change in $\Delta_{growth}\%$ was minimal, in case of Model 1 the behaviour was erratic and no specific trend was observed (Figure S4).

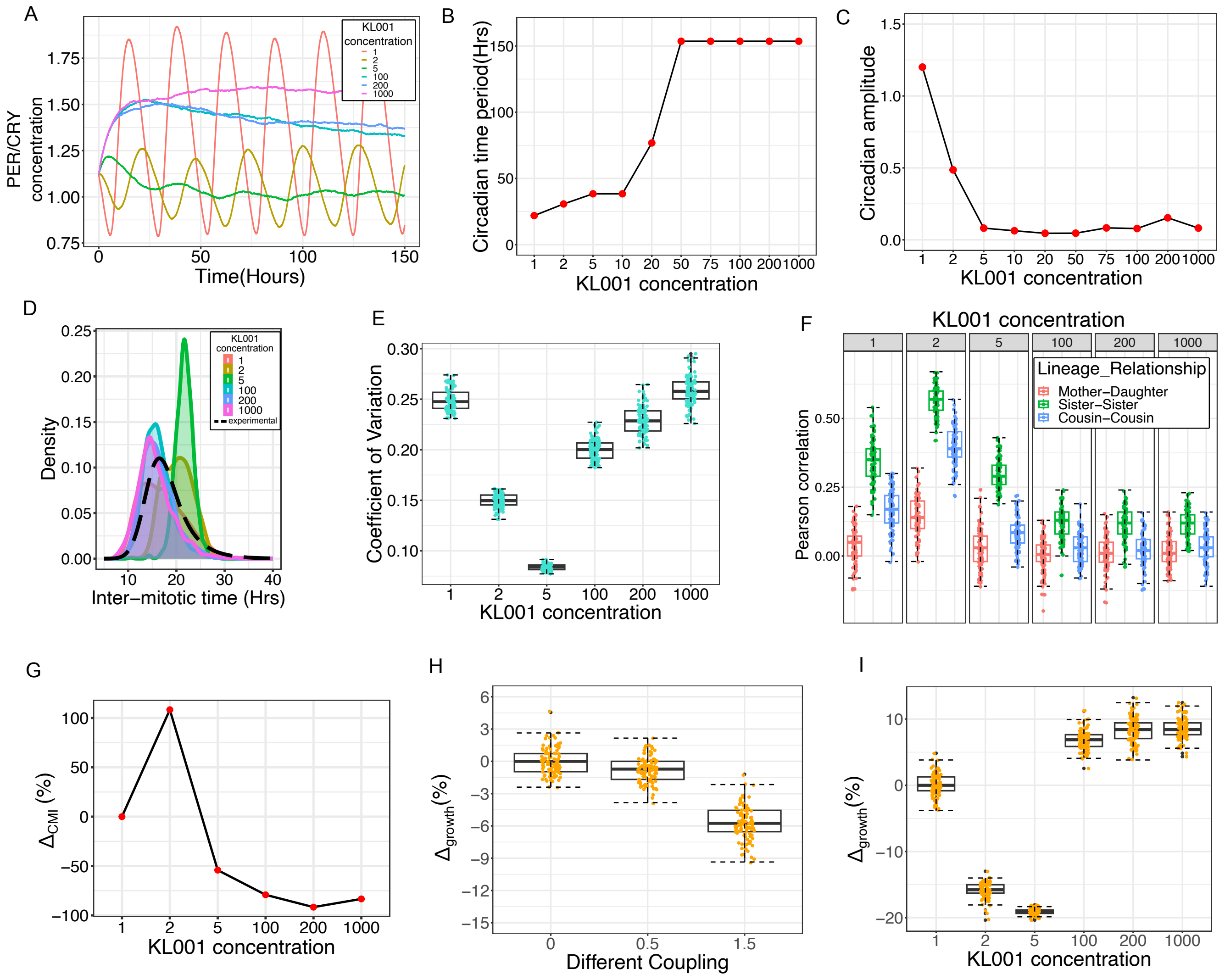

Figure S5: Circadian clock perturbation with KL001 decreases IMT correlations for Model 1: (A) Damped circadian proteins' oscillations as observed for increasing concentrations of KL001. (B) & (C) Change in time period and amplitude of circadian clock as observed under influence of KL001 (D) Changes in IMT distribution for different KL001 concentration. (E) Effect of KL001 on the Coefficient of variation of IMT distribution. While for Model 2 we observed a decrease in the variance of IMT distribution with increasing KL001, we do not observe any such trend for Model 1. (F) KL001 diminishes the cousin-mother inequality in lineage simulation similar to behviour observed for Model 2. (G) Percentage change in median value of cousin-mother inequality in comparison to the control case, shown for the different concentrations of KL001. (H) Change in population growth rate for increasing coupling strengths, predict a decrease due to increase in the cell cycle period owning to entrainment by the circadian clock. (I) Change in growth rate when KL001 addition is simulated the change in growth rate is erratic and no visible trend is present. (Boxplot and median value calculated for 100 runs of simulation. Here C = 1.5, TCC=16hrs, for the lineage simulations under KL001 effect.) (Related to Figure 3)

S9 Method S9: Digital extraction of KL001 data and downstream processing, related to Figure 3.

We obtained the luminescence rhythm data of Bmal1-dLuc and Per2-dLuc reporter U2OS, Human osteosarcoma cell line under the influence of different concentrations of KL001 from published literature [10]. Using publicly available software DataThief III [11], we digitally extracted the data points from the graphs. Having obtained a time series, we performed Fourier Transform as defined earlier in Section 4 to extract the time period (1/frequency with maximum spectral density) of oscillations for the different concentrations of KL001. Eliminating the initial transients, we also extracted the average amplitude over the different oscillatory cycles.

S10 Method S10: The Ergodic Principle and detecting M phase oscillations in cellular lineages, related to Figure 5.

To study the implementation of chronotherapy we examined the distribution of cells in different cell cycle phases in the presence and absence of circadian control. In order to do so, we generated deep lineages for 100 asynchronous ancestral cells using our lineage simulation. At every time step we recorded the proportion of cells in the different cell cycle phases. Hence according to the cell cycle model we implemented in the main text, cells were monitored across 3 different phases - G1, S/G2, and the M phase. We also recorded for each cell the absolute time at which it transitioned from one phase to another.

The fraction of cells in each cell cycle phase, in the absence of external perturbation by the clock, can be predicted by the ergodic theory. The ergodic principle states that the fraction of cells in a particular cell cycle phase is proportional to the duration of that phase. In a previous paper [12], the authors applied the ergodic principle to study the fraction of cells in each cell cycle phase. Since a cell division event leads to the disappearance of an old cell (the mother cell) but introduces two daughter cells of age 0 into the population, the fraction of cells in the G1 phase is higher than what is predicted by simple ergodicity. Correcting for the this phenomenon, the fraction of cells including and upto a phase n, $p_{\leq n}$, is given by the following expression ([12]):

$$p_{\geq n} = 2(1 - 2^{-t_{\leq n}/T}), \tag{49}$$

where $t_{\leq n}$ denotes the duration of phases up to and including phase n. This rule applies only in cases where the cell cycle does not have any external perturbations from the clock. As shown in the main text Figure 5A, the proportion of cells in our simulations matches the predicted proportion when there is no clock coupling to the cell cycle, thus validating that our simulations follow the ergodic principle.

References

[1] Raouf El Cheikh, Samuel Bernard, and Nader El Khatib. “Modeling circadian clock–cell cycle interaction effects on cell population growth rates”. In: *Journal of Theoretical Biology* 363 (2014), pp. 318–331.

[2] Daniel T Gillespie. “The chemical Langevin equation”. In: *The Journal of Chemical Physics* 113.1 (2000), pp. 297–306.

[3] Takuya Matsuo et al. “Control mechanism of the circadian clock for timing of cell division in vivo”. In: *Science* 302.5643 (2003), pp. 255–259.

[4] Xuan Zhao et al. “Circadian amplitude regulation via FBXW7-targeted REV-ERBα degradation”. In: *Cell* 165.7 (2016), pp. 1644–1657.

[5] Claude Gérard and Albert Goldbeter. “A skeleton model for the network of cyclin-dependent kinases driving the mammalian cell cycle”. In: *Interface Focus* 1.1 (2011), pp. 24–35.

[6] H Wearing. *Spectral analysis in R.* 2010.

[7] GCAT Nepusz and Gábor Csárdi. “The igraph software package for complex network research”. In: *Complex Systems* 1695.5 (2006), pp. 1–9.

[8] Gabor Csardi and Tamas Nepusz. “Igraph: Network analysis and visualization”. In: *R package* (2020).

[9] Shaon Chakrabarti et al. “Hidden heterogeneity and circadian-controlled cell fate inferred from single cell lineages”. In: *Nature Communications* 9.1 (Dec. 2018). Number: 1 Publisher: Nature Publishing Group, p. 5372.

[10] Tsuyoshi Hirota et al. “Identification of small molecule activators of cryptochrome”. In: *Science (New York, N.Y.)* 337.6098 (Aug. 2012), pp. 1094–1097.

[11] B Tummers. *DataThief III. 2006, https://datathief.org/*. 2006.

[12] Richard John Wheeler. “Analyzing the dynamics of cell cycle processes from fixed samples through ergodic principles”. In: *Molecular Biology of the Cell* 26.22 (2015), pp. 3898–3903.
